# Naïve or Engineered Extracellular Vesicles from Different Cell Sources: Therapeutic Tools for Kidney Diseases

**DOI:** 10.3390/pharmaceutics15061715

**Published:** 2023-06-12

**Authors:** Elena Ceccotti, Gabriele Saccu, Maria Beatriz Herrera Sanchez, Stefania Bruno

**Affiliations:** 1 Department of Medical Sciences, University of Torino, 10126 Torino, Italy; gabriele.saccu@unito.it; 2 Molecular Biotechnology Center, University of Torino, 10126 Torino, Italy; mariabeatriz.herrera@unito.it; 3 2i3T, Società per la Gestione dell’incubatore di Imprese e per il Trasferimento Tecnologico, University of Torino, 10126 Torino, Italy

**Keywords:** acute kidney injury, chronic kidney injury, mesenchymal stem cells, human liver stem cells, engineering

## Abstract

Renal pathophysiology is a multifactorial process involving different kidney structures. Acute kidney injury (AKI) is a clinical condition characterized by tubular necrosis and glomerular hyperfiltration. The maladaptive repair after AKI predisposes to the onset of chronic kidney diseases (CKD). CKD is a progressive and irreversible loss of kidney function, characterized by fibrosis that could lead to end stage renal disease. In this review we provide a comprehensive overview of the most recent scientific publications analyzing the therapeutic potential of Extracellular Vesicles (EV)-based treatments in different animal models of AKI and CKD. EVs from multiple sources act as paracrine effectors involved in cell-cell communication with pro-generative and low immunogenic properties. They represent innovative and promising natural drug delivery vehicles used to treat experimental acute and chronic kidney diseases. Differently from synthetic systems, EVs can cross biological barriers and deliver biomolecules to the recipient cells inducing a physiological response. Moreover, new methods for improving the EVs as carriers have been introduced, such as the engineering of the cargo, the modification of the proteins on the external membrane, or the pre-conditioning of the cell of origin. The new nano-medicine approaches based on bioengineered EVs are an attempt to enhance their drug delivery capacity for potential clinical applications.

## 1. Introduction

Synthetic drug delivery systems have been developed to enhance the pharmacodynamic and pharmacokinetic profile of therapeutics [1]. Among the most suitable ones, liposomes are lipid bilayer structures with an internal aqueous core where hydrophilic drugs can be accommodated [1]. Issues such as cytotoxicity and immunogenicity limit their application, but they can be overcome by the introduction of natural drug delivery systems, such as extracellular vesicles (EVs) [1].

EVs are heterogeneous phospholipid bilayer-enclosed nanoparticles that are classified into three categories according to their biogenesis: apoptotic bodies (50 nm–5 µm), microvesicles (50 nm–1 µm), and exosomes (30 nm–150 nm) [1]. They transport active biomolecules and act as mediators of cell-to-cell communication in many pathophysiological processes [2]. EVs are comparable to liposomes because both are phospholipid-based structures, but EVs are packed in a cell-specific way and they show distinctive properties over synthetic vehicles [1,2]. Considering their biological origin, natural vesicles are biocompatible and less immunogenic than synthetic systems [2]. EVs are also safe drug carriers because, different from virus-derived structures, they are non-replicative and not mutagenic [1]. Another important feature of EVs is their ability to cross biological barriers, including the blood-brain barrier, and through endocytosis, they are internalized in the recipient cell to induce a response [1,2]. While liposomes passively accumulate in certain organs, EVs have an intrinsic targeting ability which enhances the delivery of bioactive molecules in the desired site [1,2].

Proteins are abundant in the EV cargo and they vary according to the cell of origin, the physiological state, and the route of biogenesis [3]. The proteins belonging to the endosomal sorting complex for transport (ESCRT) machinery and Alix play a crucial role in their biogenesis and are enriched compared to the cell of origin [1]. Apart from proteins, coding and noncoding RNAs, such as mRNA, miRNA, and circRNA, are loaded in EVs [1]. RNA sorting is regulated by an organized mechanism based on specific sequence motifs which bind to RNAs and enhance their loading into the vesicles [1]. 

EVs are emerging as a promising cell-free strategy for the treatment of acute kidney injuries (AKI) and chronic kidney diseases (CKD) [4,5] and could represent a next generation nano-medicine approach to treat renal pathologies [5,6]. In this review, we will provide an overview of the therapies based on EVs in animal models of AKI and CKD and we will compare the effects of the EV populations isolated from different cell sources. Moreover, we will discuss the application of bioengineered EVs as a new option for the treatment of kidney diseases.

## 2. EVs of Different Origins for the Treatment of Kidney Diseases 

In recent years, stem cell therapies have been used in regenerative medicine and tissue engineering for the treatment of kidney diseases [7]. Mesenchymal stromal cells (MSCs) are among the most studied cells due to their multipotent cell differentiation capacity and self-replenishment [8]. MSCs can be isolated from different tissues, such as bone marrow, adipose tissue, umbilical cord, muscles, and placenta, and they can differentiate into multiple cell types according to external conditions [8]. These cells have anti-inflammatory properties as shown by their ability to be recruited in the injurious site and to secret growth factors; they are also associated with side effects, such as immune rejection and decreased proliferation capacity over time [8]. 

**Figure 1:**
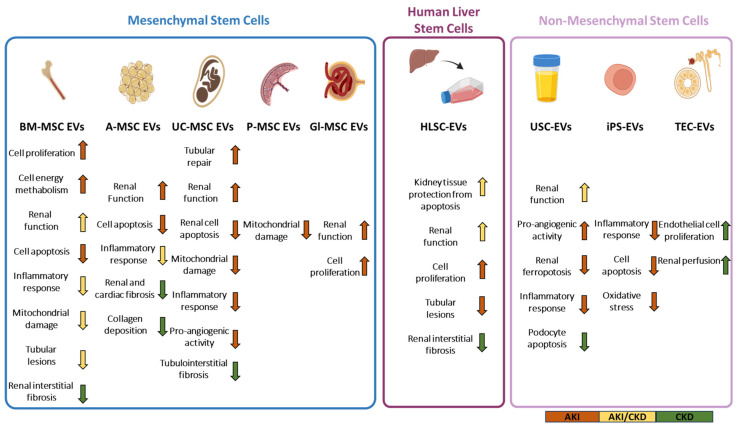
Different cell sources for the isolation of EVs used for the treatment of kidney diseases and their mechanisms of action. This picture summarizes the main cell sources of the EVs used for the treatment of kidney diseases and their principal therapeutic effects in AKI (orange arrows), AKI-CKD transition (yellow arrows), and CKD (green arrows).

Growing evidence has demonstrated that the therapeutic effects of MSCs are mediated by their secretome, where MSC-EVs play a major role [4,9]. MSC-EVs show low immunogenicity and tumorigenicity with respect to the cell of origin [10]. Different studies analyzed the therapeutic potential of MSC-EVs in various models of AKI and CKD, and most of them are concentrated on the application of EVs isolated from the bone marrow (BM-MSCs), the adipose tissue (A-MSCs) and the umbilical cord (UC-MSCs) [4] (Figure 1). 

Interestingly, another promising source of EVs is represented by urine stem cells (USC-EVs) [11,12] (Figure 1). They may have a stronger regenerative capacity in the injured kidney due to their intrinsic renal commitment [11]. 

Human liver stem cells (HLSCs) represent a population of stem cells obtained by the human adult liver which share some properties with MSCs, such as the same phenotype, gene expression profile, and differentiation capabilities [13] (Figure 1). HLSC-EVs showed pro-regenerative and tissue-protective properties not only in the liver but also in the kidney [14].

Alternatively, therapeutic EVs can be isolated from induced pluripotent stem cells (iPSC) [4] (Figure 1). iPSCs are induced to differentiate into MSCs, but differently from other MSC sources, iPSC-MSCs show an indefinite proliferative capacity [4]. 

## 3. Extracellular Vesicles Applications in AKI

AKI represents a global health issue associated with elevated morbidity and mortality and is a clinical complication of hospitalized patients [15,16]. AKI is caused by different insults such as sepsis, ischemia, nephritis, systemic inflammation, oxidative stress, nephrotoxic medications, environmental pollutants, and urinary tract obstructions [17]. There is an imbalance in the local tissue supply of oxygen and the accumulation of metabolic waste products [16]. This condition results in tubular epithelial cells injury and death, leading to functional impairment of water and electrolyte homeostasis [16]. The onset of AKI is clinically characterized by increased plasma levels of creatinine and blood urea nitrogen (BUN) [18]. The functional unit of the kidney, the nephron, is mainly interested in the pathophysiology of AKI; the renal tubular epithelial cells undergo inflammatory and oxidative stresses resulting in cell death, vascular failure, and altered functionality [16,17]. 

Several studies have examined the potential therapeutic role of EVs in animal models of AKI induced by ischemia-reperfusion injury (IRI), and injection of glycerol or cisplatin, which were further discussed in the following sections.

### 3.1. MSC-Derived EVs in AKI

A single administration of BM-MSC-EVs promoted recovery of renal function and morphology by increasing tubular cell proliferation in a murine model of glycerol-induced AKI [19] (Table 1) (Figure 1). In addition, in a cisplatin-AKI model, a single injection of BM-MSC-EVs protected the renal tubular cells from apoptosis resulting in the enhancement of mouse survival due to improved kidney function and histology [20]. The protective effect of EV injection was also reported in a single-dose treated rat model of renal IRI [21]. After reperfusion, EVs promptly inhibited tubular cell apoptosis and enhanced their proliferation [21]. BUN and Creatinine plasma levels were ameliorated following EV treatment [21]. In these papers, it has been shown that EVs pretreated with RNase have a reduced protective effect on renal morphology and function, indicating the involvement of EV-RNAs in the modulation of their biological effects on target cells [19,20,21].

The EV miRNA content plays a crucial role in kidney repair after injury by regulating cell death and proliferation [22]. miR-125b-5p/p53, which is involved in the p53 pathway, was carried by human UC-MSC-EVs and promoted tubular repair by regulating the cell cycle and reducing apoptosis in a mouse model of ischemic AKI [23]. Moreover, miR-199a-3p carried by BM-MSC-EVs protected the kidney against IRI by modulating Akt and Erk1/2 signaling pathways [24].

In a cisplatin-induced AKI rat model, the injection of UC-MSC-EVs under the renal capsule improved kidney function by reducing oxidative stress and cell apoptosis, as well as by fostering cell proliferation [25]. 

Another important aspect to take in consideration in AKI is the inflammation. Very interestingly, the inflammatory response triggered by AKI was modulated by MSC-EVs through the transport of anti-inflammatory molecules [26]. In a sepsis-induced AKI mouse model, the administration of UC-MSC-EVs showed a therapeutic effect by modulating inflammation through the inhibition of the NF-kB pathway induced by miR-140b up-regulation [27]. In a porcine model of AKI, h-UC-MSC-EVs further showed anti-inflammatory capabilities by reducing STAT3 and NF-kB signaling pathways and increasing the expression of reno-protective molecules, such as bone morphogenetic protein 7 (BMP7) and Klotho [28]. In addition, CX3CL1 carried by UC-MSC-EVs modulated renal macrophage accumulation in a rat model of IRI-AKI [29]. The anti-inflammatory activity was also observed injecting A-MSC-EVs, which modulated Sirtuin 1 (SIRT1) signaling pathway [30]. Indeed, A-MSC-EV administration inhibited kidney inflammation and apoptosis in sepsis-induced AKI [30].

In a lipopolysaccharide (LPS)-induced AKI rat model, both BM-MSC-EV and A-MSC-EV treatment improved renal structure and function by down-regulating oxidative stress and inflammation, as well as influencing the kidney autophagy levels [31].

Recent studies have shown that MSC-EVs also modulated angiogenesis in AKI [32]. UC-MSC-EV treatment in a unilateral IRI rat model enhanced the pro-angiogenic activity in a hypoxia-inducing factor-1α (HIF-1α)-independent manner [32]. 

Mitochondrial (mt) damage is recognized as an injurious stimulus for AKI and MSC-EVs can regulate mitochondrial transcriptional factor A (TFAM) and mt-DNA delivery [33]. BM- and h-UC-MSC-EVs both attenuated renal dysfunction, and mitochondrial damage and down-regulated the inflammatory response in an IRI mouse model [33]. Mitochondrial function was also regulated by human Placenta MSC-EVs (h-P-MSC-EVs) in a glycerol-induced AKI mouse model [34]. 

Noteworthy, Gregorini M. et al. demonstrated the significant role of BM-MSC-EVs in the kidney donor circulatory death (DCD) rat model [35]. EV treatment induced the reduction of the ischemic damage and the up-regulation of cell energy metabolism thus stimulating cell viability [35]. 

In the kidney, it has been shown the presence of a pool of stem cells, such as Glomerular-MSC (Gl-MSCs), which represent a potential source of EVs with protective capabilities as demonstrated in a mouse model of IRI-induced AKI [36]. Gl-MSC-EVs preserved the kidney from acute induced damage, due to their miRNA cargo which may contribute to the functional and structural recovery of the kidney [36].

Furthermore, BM-MSC-EV administration proved their capabilities in organ preservation IRI strategy in ex vivo hypothermic oxygenated perfusion in marginal kidneys from expanded criteria donors (ECD) [37]. Thus, MSC-EV treatment not only improved the renal regeneration in different in vivo models but also represented a strategy for increasing the pool of donors and enhancing the transplant outcome [37].

**Table 1:** MSC-EVs in AKI.

In Vivo Model	EV-Source	EV Isolation Method	EV Route of Administration	EV Biological Effect/ Mechanism of Action	References
**Glycerol mouse model** Intra-muscular glycerol injection into inferior hind limbs (8 mL/kg)	BM-MSCs (15 µg/dose)	UC	Single intravenous injection 3 days after the damage	- Stimulation of renal cell proliferation	[19]
**Cisplatin mouse model** Subcutaneous cisplatin injection (12 mg/kg)	BM-MSCs (100 µg/dose)	UC	Single injection 8 h after the damage	- Reduction of apoptosis	[20]
**IRI rat model** Ligation of the left renal artery and vein for 45 min with contralateral nephrectomy	BM-MSCs (30 μg/dose)	UC	Single intravenous injection immediately after surgery	- Inhibition of renal cell apoptosis and stimulation of their proliferation - Amelioration of renal function	[21]
**IRI mouse model** Renal pedicle clamping for 30 min	h-UC-MSCs (100 µg/dose)	UC	Two intravenous injections after IRI	- miR-125b-5p/p53, promoted tubular repair targeting cell cycle and reduced apoptosis	[23]
**IRI mouse model** Renal pedicle clamping for 45 min	BM-MSCs (5 × 10^10^ EV/dose)	UC	Single tail intravenous injection 1 h before IRI	- miR-199a-3p protected the kidney by modulating Akt and Erk1/2 pathways	[24]
**Cisplatin rat model** Single intraperitoneal cisplatin injection (6 mg/kg)	UC-MSCs (200 µg/dose)	UC	Single injection in both the renal capsules 24 h damage	- Reduction of oxidative stress and apoptosis - Stimulation of cell proliferation	[25]
**Sepsis mouse model** Cecal ligation and puncture with CLP	UC-MSCs (120 μg/dose)	UC	Tail intravenous injection 3 h after CLP injury	- miR-140b up-regulation inhibited NF-kB pathway - Modulation of inflammation	[27]
**IRI porcine model** Unilateral ischemia for 120 min and contralateral nephrectomy	UC-MSCs (1 × 10^9^ EV/dose)	UC and sequential 100 K Amicon centrifugal filter	Intravenous injection during the ischemia	- Reduction of STAT3 and NF-kB pathways - Expression of KLOTHO and BMP7	[28]
**IRI rat model** Left renal ischemia for 60 min	UC-MSCs (100 μg EV/dose)	UC	Caudal intravenous injection immediately after reperfusion	- CX3CL1 modulates macrophage accumulation	[29]
**Sepsis mouse model** Cecal ligation and puncture with CLP	A-MSCs (100 μg EV/dose)	UC	Tail intravenous injection	- Inhibition of inflammation and apoptosis - Modulation of the SIRT1 signaling pathway	[30]
**LPS rat model** Intraperitoneal injection of LPS (7.5 mg/kg)	BM-MSCs and A-MSCs (5 × 10^5^ EV/dose)	UC	Single Injection 30 min before LPS injection	- Improved renal function and structure - Down-regulation of oxidative stress and inflammation	[31]
**Unilateral IRI mouse model** Renal artery clamping for 45 min	UC-MSCs (100 μg EV/dose)	UC	Caudal vein injection immediately after reperfusion	- Enhancement of pro-angiogenic activity in a HIF-1α-independent manner	[32]
**IRI mouse model** Bilateral renal pedicle clamping for 30 min	BM-MSCs and UC-MSCs (6.96 × 10^10^ EV/dose)	UC	Single-tail intravenous injection	- Reduction of mitochondrial damage - Down-regulation of inflammatory response	[33]
**Glycerol mouse model** Intramuscular glycerol injection into inferior hind limbs (8 mL/kg)	P-MSCs (80 µg EV/dose)	UC	Single tail intravenous injection 3 days after damage	- Modulation of mitochondrial damage	[34]
**Rat kidney DCD ex vivo model**	BM-MSCs	UC	Ex vivo EV perfusion	- Reduction of ischemic damage - Up-regulation of cell energy metabolism	[35]
**IRI mouse model** 35 min Left renal pedicle clamping for 35 min and contralateral nephrectomy	Gl-MSCs (400 × 10^6^ EV/dose)	UC	Single tail intravenous the administration immediately after surgery	- Recovery of renal function and structure	[36]

Abbreviations: Ultracentrifugation (UC), Ischemia and reperfusion injury (IRI), bone marrow mesenchymal stromal cells (BM-MSCs), human umbilical cord-MSCs (hUC-MSCs), adipose MSCs (A-MSCs), lipopolysaccharide (LPS), placental-MSCs (P-MSCs), glomerular MSCs (Gl-MSC), donor circulatory death (DCD).

### 3.2. Human Liver Stem Cell (HLSC)-Derived EVs in AKI

Human liver stem cells-derived EVs (HLSC-EVs) have been evaluated as an alternative cell-free strategy to treat AKI. HLSC-EVs showed a pro-regenerative effect in a glycerol-induced AKI mouse model and in vitro on murine tubular epithelial cells (mTECs) [38]. In this study, the administration of two different EV doses improved renal function after damage, indeed plasma levels of BUN and Creatinine were decreased. Moreover, both doses enhanced kidney proliferation rate and reduced tubular necrosis [38]. Likewise, the renoprotective role of EVs was evaluated and confirmed in vitro, where EVs induced the proliferation of TECs and inhibited their apoptosis [38]. Interestingly, the role of HLSC-conditioned medium (HLSC-CM) was also evaluated. The HLSC-CM was more effective in vivo than the CM depleted of EVs, showing the relevant role of vesicles in renal regeneration [38].

### 3.3. Non-MSC-Derived EVs in AKI

The efficacy of EVs derived from USCs, h-iPS and urine have been evaluated in models of AKI (Table 2).

The iPSC-EVs have a similar therapeutic potential to A-MSC-EVs in a rat model of IRI-AKI [39]. Indeed, both the EV treatments supported tissue recovery by down-regulating inflammation, reducing cell death, and protecting mitochondria from oxidative stress [39]. 

Treatment with USC-EVs improved renal function in a lethal rat model of IRI-AKI [40]. EV injection reduced apoptosis inhibited the inflammatory response and decreased the plasma levels of BUN and creatinine [40]. USC-EV shuttled some miRNAs, such as miR-146a-5p, which were able to counteract the infiltration of inflammatory cells by down-regulating interleukin-1 receptor-associated kinase 1 (IRAK1) and protecting the kidney from IRI [40]. In addition, USC-EVs played a role in IRI-AKI by regulating Achaete-Scute Family BHLH Transcription Factor 4 (ASCL4)-ferroptosis via lncRNA-TUG1 [41]. In a murine model of glycerol-induced AKI, the EVs obtained from the urine (uEVs) of healthy voluntaries induced renal recovery by stimulating TEC proliferation and downregulating pro-inflammatory markers [42]. Interestingly, uEVs shuttled Klotho, a renoprotective molecule, which mediated the therapeutic potential of this EV population [42].

## 4. Extracellular Vesicles Application in CKD 

**Figure 2:**
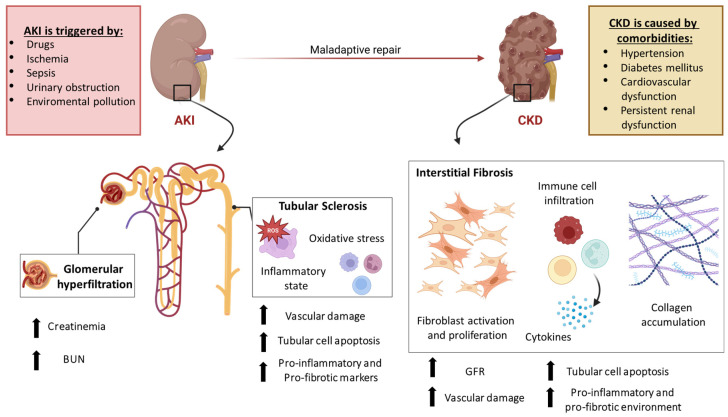
Pathophysiological features of AKI-CKD transition. The upper part of the cartoon reports the main causes inducing AKI and those contributing to the maladaptive repair which predisposes to CKD. In the lower part of the cartoon, the main functional and morphological kidney damages are illustrated. Arrows indicate the increase of the pathophysiological alterations related to AKI and CKD development.

Multiple pathophysiological alterations occur in association with AKI such as glomerular hyper-filtration, proteinuria, tubular sclerosis, and interstitial inflammation leading to the progressive decline of renal function and morphology [43]. Considering the severity of the damage and the incidence of other health problems there is an increased risk of a maladaptive repair after AKI which predisposes to the progression to CKD [44] (Figure 2).

CKD is a global medical problem whose development is mainly associated with comorbidities such as hypertension, diabetes mellitus, cardiovascular, and renal dysfunction [43]. Interstitial fibrosis, which consists of the proliferation of resident fibroblasts and the accumulation of extracellular matrix (ECM) components, is a hallmark of CKD, and leads to the progressive deterioration of kidney structure and function ultimately leading to an end-stage condition [45,46]. 

Over the past years, several research groups demonstrated the therapeutic effects of EVs from different sources in animal models of CKD, such as aristolochic acid induce nephropathy (AAN), diabetic nephropathy (DN), unilateral ureteral obstruction (UUO), IRI, and 5/6 partial subtotal nephrectomy (PNx) (Figure 1).

### 4.1. MSC-Derived EVs in CKD

Administration of a single dose of h-BM-MSC-EVs immediately after IRI not only improved AKI recovery but also prevented CKD development [21] (Table 3). IRI rats treated with EVs showed lower plasma levels of BUN and creatinine with respect to untreated animals and they also showed less interstitial lymphocyte infiltrates and tubular atrophy [21]. It has been also reported that multiple administrations of BM-MSC-EVs ameliorated kidney function and tubular atrophy in mice surviving to AKI-cisplatin injury [20]. 

DN is a diabetes-related complication, characterized by glomerular and tubulointerstitial fibrosis; it is a risk factor associated with the onset of CKD [46]. The administration of multiple doses of h-BM-MSC-EVs in a murine model of streptozotocin (STZ)-induced CKD prevented the progression of DN and subsequently prevented renal glomerular and interstitial fibrosis. EV-treated mice showed a down-regulation of pro-fibrotic markers [46]. 

AAN has been recently recognized as a cause of CKD and AA is a toxin presents in plants used for herbal therapy and its constant assumption can lead to the development of progressive renal diseases until end-stage organ failure [47,48]. AAN is characterized by tubular damage, interstitial fibrosis, and urothelial malignancies in 40% of the cases [48]. The repeated injections of h-BM-MSC-EVs in the AAN model significantly ameliorated kidney tissue histology by reducing interstitial fibrosis and infiltration of inflammatory cells [49]. The EV treatment significantly reduced the plasma levels of BUN and down-regulated the expression of the most common pro-fibrotic markers Alpha Smooth Muscle Actin (α-Sma) and Collagen 1a1 (Col1a1) [49]. 

Wang Y. et al. reported the potential therapeutic effects of rat BM-MSC-derived EVs in a rat model of UUO-induced CKD [50]. UUO rats showed increased plasma levels of BUN associated with histological changes, including tubular dilatation and necrosis, and increased ECM deposition in the tubulointerstitium [50]. The injection of a preventive single dose of BM-MSC-EVs significantly reduced the tubular lesions, the structural damage in the renal parenchyma, and the blood levels of BUN [50]. At the molecular level, the EV treatment down-regulated the expression of the pro-fibrotic marker α-SMA and increased the E-cadherin-positive areas [50]. 

Similar results were obtained in another rat model of UUO-induced CKD where a single dose of rat BM-MSC-EVs was administered [45]. BM-MSC-EVs decreased the deposition of collagen protein and interestingly, preserved the physiological amount of renal CD34+ cells, an index of intact microvasculature [45]. Shi Z. et al. investigated the molecular mechanisms involved in the therapeutic potential of rat BM-MSC-EVs and they highlighted the presence of a protein in the bioactive cargo of this EV population: the milk fat globule–epidermal growth factor–factor 8 (MFG-E8), known also as lactadherin [45]. It is a secreted multifunctional glycoprotein involved in the attenuation of oxidative stress and recent evidence underlines its ability to regulate the RhoA/ROCK signaling pathway which is induced by TGF-β1 [45]. MFG-E8 carried by BM-MSC-EVs was able to reduce renal fibrosis in UUO-treated rats by inhibiting the RhoA/ROCK signaling pathway, and the biological effects of these EVs were abrogated by silencing MFG-E8 [45].

He J. et al. tested the therapeutic potential of mouse BM-MSC-EVs in a murine model of UUO-induced CKD and they found 81 up-regulated miRNAs in the EV bioactive cargo, some of them are associated with the EMT [51].

Cheng Ji et al. proposed a different therapeutic strategy in a rat model of UUO-induced CKD by administering EVs derived from h-UC-MSC-EVs [52]. In this work, the researchers reported an increased expression of the Yes-associated protein (YAP) in UUO rats; this protein is a co-factor of the Hippo pathway, and its dysregulation contributes to the progression of renal fibrosis [52]. YAP activation increased the deposition of ECM components which further activate YAP expression creating a positive loop, but the administration of a single dose of h-UC-MSC-EVs was able to induce YAP ubiquitination and degradation [52]. Two components of the ubiquitination system, casein kinase 1δ (CK1δ) and E3 ubiquitin ligase β-TRCP were enriched in h-UC-MSC-EVs and they contributed to the inhibition of YAP activity leading to the reduction of tubulointerstitial fibrosis and subsequent amelioration of kidney fibrosis [52]. 

In the PNx model, the predominant pathological abnormalities consist of glomerular sclerosis and interstitial fibrosis that recapitulate the renal dysfunction of patients with CKD [53]. BM-MSC-EV administration slightly reduced BUN levels in the blood, but on the other hand, there was a significant reduction of serum creatinine and an amelioration of proteinuria [54]. At the histological level, the EV treatment improved kidney histology by reducing tubular swelling and necrosis, interstitial lymphocyte infiltrates, the formation of cast zones, and the deposition of collagen [54]. 

In a rat model of PNx-CKD, Wan F. et al. provided insight into the mechanism of action of rat BM-MSC-EVs [55]. This EV population effectively ameliorated renal pathological features by upregulating the expression of Klotho, an anti-aging gene dysregulated by CKD progression [55].

Administration of h-ASC-EVs led to multiorgan beneficial effects in a deoxycorticosterone acetate (DOCA)-salt hypertensive rat model [56]. In this study, the high-salt diet was combined with unilateral nephrectomy drastically increasing the onset of hypertensive-induced CKD [56]. The DOCA is an aldosterone precursor which increases the Na+ levels and the reabsorption of water by the distal nephron, this condition promotes hypervolemia-induced hypertension [56]. Multiple injections of h-ASC-EVs prevented renal functional alterations and the progression of fibrosis both in the kidney and in the heart, where the EVs contributed also to maintain normal blood pressure [56]. The researchers analyzed the miRNA profile of DOCA-salt rats and identified a peculiar EMT miRNA signature induced by DOCA treatment, which increased the expression of the TGF-β gene [56]. ASC-EVs altered one of the key pathways, the miR-200-TGF-β axis, preventing the inflammatory response and the development of fibrosis in the kidney [56]. 

**Table 3:** MSC-EVs in CKD.

In Vivo Model	EV-Source	EV Isolation Method	EV Route of Administration	EV Biological Effect/ Mechanism of Action	References
**IRI rat model** 45 min IRI and contralateral nephrectomy	h-BM-MSCs (30 µg EV/dose)	UC	Single intravenous injection immediately after IRI	- Reduced plasma levels of BUN and creatinine - Reduced lymphocyte infiltrates and tubular atrophy	[21]
**Cisplatin mouse model** Subcutaneous cisplatin injection (12 mg/kg)	BM-MSCs (100 µg EV/dose; 50 µg EV/dose)	UC	First (100 µg/dose) intravenous injection 8 h after the damage. Multiple (50 µg/dose) injections 2, 6, 10, 14, and 18 days after the damage	- Reduced tubular damage - Reduced plasma levels of BUN and creatinine	[20]
**STZ-DN mouse model** Intraperitoneal injection of STZ (37 mg/kg) for 4 consecutive days	h-BM-MSCs (1 × 10^10^ EV/dose)	UC	Multiple intravenous injections once a week for 4 weeks starting from day 30 after diabetes onset	- Reduced renal fibrosis - Reduced plasma levels of BUN and creatinine - Reduced expression of pro-fibrotic and pro-inflammatory genes	[46]
**AAN mouse model** Intraperitoneal injection of 4 mg/kg of AA on a weekly basis for 4 weeks	h-BM-MSCs (1 × 10^10^ EV/dose)	UC	Multiple intravenous injections on a weekly basis starting from 3 days after AA administration	- Reduced tubular necrosis - Reduced interstitial fibrosis - Reduced inflammatory infiltrates - Down-regulation of pro-fibrotic genes	[49]
**UUO rat model** Ligation of the left ureter and the right the ureter was not ligated	Rat-BM-MSCs (3 × 10^5^ EV/dose)	UC and exoEasy Maxi Kit (Qiagen, Germany)	Single tail vein injection 3 days before UUO surgery	- Reduced BUN plasma level - Reduced tubular lesions - Reduced α-SMA and increased E-cadherin expression	[50]
**UUO rat model** Ligation of the left ureter and the right the ureter was not ligated	Rat-BM-MSCs (0.5 mg/kg EV/dose)	UC	Single intravenous injection 1 day after UUO surgery	- Reduced renal fibrosis, inflammation and oxidative stress - MFG-E8 inhibited the RhoA/ROCK signaling pathway	[45]
**UUO mouse model** Ligation of the left ureter and the right the ureter was not ligated	m-BM-MSCs (30 mg EV/dose)	UC	Single caudal vein injection 2 days after the UUO surgery	- Reduction of tubulointerstitial fibrosis - Reduced plasma levels of creatinine	[51]
**UUO rat model** Ligation of the left ureter and the right the ureter was not ligated	h-UC-MSCs (200 µg EV/dose)	UC	Not specified	- CK1δ and β-TRCP inactivated YAP activity - Reduction of tubulointerstitial fibrosis	[52]
**PNx mouse model** Resection of the upper and lower pole of the left kidney. Contralateral nephrectomy one week after	m-BM-MSCs (30 mg EV/dose)	UC	Multiple caudal vein injections 2, 3, and 5 days after the surgery	- Reduced plasma levels of BUN and creatinine - Reduced tubular necrosis - Reduced lymphocyte infiltrates - Reduced collagen deposition	[54]
**PNx rat model** Resection of the upper and lower pole of the left kidney. Contralateral nephrectomy one week after	r-BM-MSCs (1 × 10^7^ EV/dose)	UC	Multiple caudal vein injections on days 30 and 45 post surgery	- Amelioration of renal function and histology - Increased expression of Klotho	[55]
**DOCA rat model** Nephrectomy of the left kidney. One week after the animals started a high-Na+ diet with subcutaneous DOCA	h-ASCs (1.5 × 10^9^ EV/dose + DMSO 0.2 mg/~300 g)	UC	Weekly intravenous injection starting from 2 weeks after nephrectomy	- Reduced inflammatory response and fibrosis both in the kidney and in the heart - Reduced inflammatory response	[56]

Abbreviations: ultracentrifugation (UC), ischemia and reperfusion injury (IRI), bone marrow mesenchymal stromal cells (BM-MSCs), adipose stem cells (ASCs), umbilical cord MSCs (UC-MSCs), streptozotocin diabetic nephropathy (STZ-DN), Aristolochic acid nephropathy (AAN), unilateral ureteral obstruction (UUO), partial 5/6 nephrectomy (PNx), and Deoxycorticosterone acetate (DOCA).

### 4.2. HLSC-Derived EVs in CKD

In the cargo of HLSC-EVs, there are bioactive molecules, in particular miRNAs, targeting genes involved in pro-fibrotic pathways such as TGF-β, epidermal growth factor receptor (EGFR), platelet-derived growth factor receptor (PDGFR) and vascular endothelial growth factor (VEGF) [46] (Table 4) (Figure 1).

The biological effects of HLSC-EVs were compared with h-BM-MSC-EVs in a DN mouse model [46]. Both the EV populations were effective in protecting the kidney in DN mice. A specific miRNA signature was detected, and bioinformatic analysis revealed the presence of common molecular targets, as TGF-β, EGFR, and PDGFR, of miRNA shuttled by the two EV-populations [46].

In addition, in an AAN mouse model, HLSC-EVs ameliorated kidney function, and morphology and reduced the expression of the pro-fibrotic markers α-Sma and Col1a1 as previously observed with h-BM-MSC-EV. Interestingly, the analysis of the miRNA profile in murine renal tissue revealed that 28 miRNAs were regulated by HLSC-EV administration and some of them seemed to target genes involved in pro-fibrotic pathways, such as the WNT signaling pathway [57]. 

In a murine model of IRI-induced AKI which subsequently developed into CKD, two intravenous injections of HLSC-EVs interfered with CKD development by effectively reducing interstitial fibrosis, the infiltration of inflammatory cells, and by down-regulating the gene expression levels of pro-fibrotic and pro-inflammatory markers, such as α-Sma, Col1a1, Tumor Necrosis Factor-α (TNF-α) and interleukin-6 (IL-6) [44].

### 4.3. Non-MSC-Derived EVs in CKD

TEC-derived EVs functions as an active vector playing an essential role in the pathogenesis of kidney disease [58] (Table 5) (Figure 1). Persistent ischemic and toxic insults impair the regenerative capacity of TECs and this leads to incomplete tubular repair which is a risk factor for the microvasculature [58]. The microvasculature damage may lead to the rarefaction of the peritubular capillaries (PTC) which results in renal hypoxia [58]. TECs maintained in hypoxic conditions increased the secretion of endothelial growth factor-A (VEGF-A) shuttled by EVs [58]. TEC-derived EVs carrying VEGF-A-induced PTC proliferation improve renal perfusion, and impair the AKI-CKD transition [58]. 

The therapeutic properties of EVs secreted from h-USC-EVs were investigated in a rat model of STZ-induced DN [59]. In this model, h-USC-EVs injection effectively ameliorated renal function by inducing the proliferation of CD31+ endothelial cells and the reduction of urinary excretion of microalbumin [59]. Through caspase-3 suppression, the EV treatment protected podocytes from apoptosis [59]. Speculating about the possible mechanism of action, the researchers highlighted that the biological cargo of h-USC-EVs included some factors, such as bone morphogenetic protein-7 (BMP-7), TGF-β1, angiogenin, and VEGF, which allowed to explain the EV ability to promote cell survival and vascular regeneration [59]. 

## 5. Engineering Strategies to Enhance the Capabilities of Native EVs

**Figure 3:**
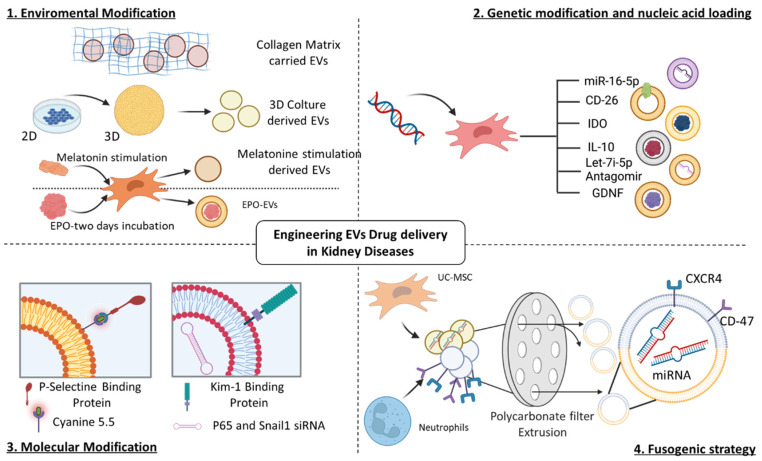
Engineering extracellular vesicles for drug delivery in kidney diseases. Schematic representation of different EV engineering methods. The main strategies include (**1**) environmental modification, (**2**) genetic modification and nucleic acid loading in the cells of origin (pre-loading strategy), (**3**) molecular modification of isolated EVs (post-loading strategy) and (**4**) fusogenic strategy.

In recent years, EVs have been described as a valuable resource for the treatment of AKI and CKD in different preclinical models. To enhance their therapeutic application, engineering strategies have been proposed to optimize and emphasize the renoprotective capacity of EVs. Among those strategies there are some concentrated on the improvement of the EV homing and innate targeting capacity of the injured organs by modifying the EV surface. Others aimed to improve the delivery of specific active molecules, such as proteins and/or miRNAs, by loading the molecules of interest inside the EVs, or extending their bioavailability in the damaged area by increasing their stability (Figure 3). To optimize these strategies, two approaches have been proposed: the engineering of the cell of origin (pre-loading approaches) or of the already isolated EV population (post-loading approaches) [60].

### 5.1. Engineering EVs for AKI Treatment 

One approach to increase the targeting of the injured kidney is the expression of the receptor for a specific endothelial biomarker of AKI, such as the P-selectin. Recently, h-P-MSC-EVs were engineered with P-Selectin binding Peptide (PBP-EVs) through a hydrophobic insertion strategy, and they were injected in IRI-AKI mice [61] (Table 6) (Figure 3). PBP-EVs preferentially targeted the injurious organ and exerted a nephron-protective function by alleviating the inflammatory infiltrates, increasing angiogenesis, and repairing renal parenchyma [61]. For those promising results, PBP-EVs may become a potential theragnostic strategy to provide a prompt diagnosis for early-stage AKI [61].

An interesting approach to enhance the targeting capacity of EVs is the creation of hybrid fused structures (Figure 3). In a recent paper it has been reported that the membrane of h-UC-MSC-EVs was fused with human neutrophil membranes, producing a hybrid vesicle called NEX [62]. These hybrid structures were injected in a mouse model of cisplatin-AKI and they reduced the recruitment of inflammatory cytokines and immune infiltrates, and promoted renal proliferation by inhibiting apoptosis [62]. Compared to naïve EVs, NEX showed an improved capacity for reaching the damaged renal tissue and the expression of CD47+ on their membrane inhibited their uptake by macrophages [62].

Transfection with plasmids or with adenoviral vectors may represent possible tools to modify the EV cargo (Figure 3). Du J. et al., transfected TECs with an adenovirus to induce CD26 overexpression and they isolated CD26+ EVs (Exo-CD26+) [63]. The injection of Exo-CD26+ in IRI-AKI mice led to a reduction of the infiltration of inflammatory cells due to the modulation of the chemokine receptor CXCR4 [63]. 

In another IRI-AKI model, BM-MSC-EVs were isolated from cell cultures transfected with plasmids to induce overexpression of Indoleamine 2,3-Deoxygenase (IDO) [64]. BM-MSC-EVs overexpressing IDO showed enhanced anti-inflammatory properties compared to natural EVs and they ameliorated AKI recovery by reducing apoptosis, inflammation, and macrophage polarization [64].

Moreover, RAW macrophages were transfected with a plasmid coding for murine interleukin 10 (IL-10) and produced IL-10+ EVs [65]. Treatment with this EV population counteracted IRI-AKI by regulating macrophage infiltration and preventing AKI-CKD transition [65]. In addition, mTOR activity was suppressed and consequently, mitophagy was promoted to maintain mitochondrial fitness in TECs [65].

Recently, red blood cells have been proposed as a new source of EVs. Red blood cell-derived EVs (REVs) were engineered with kidney injury molecule-1 (Kim-1) bound to LTH peptide (REV-LTH) [66]. REV-LTH efficiently localized at the renal injured tubules after IRI-AKI [66]. The transcription factors P65 and Snai1 were involved in the induction of the pro-inflammatory and pro-fibrotic responses. siRNAs targeting P6 and Snai1 were loaded into REV-LTH and they were effectively delivered to IRI- and UUO-damaged kidneys resulting in the alleviation of tubulointerstitial inflammation and preventing AKI-CKD transition [66]. 

Another strategy may be represented by the combination of naïve EVs with a valid matrix for supporting their therapeutic potential (Figure 3). In an IRI-AKI mouse model, h-P-MSC-EVs were injected in a collagen matrix (collagen-EVs) by improving their stability and retention in the injurious site compared to EVs alone [67]. Thus, collagen-EVs stimulated kidney proliferation, pro-angiogenic, and anti-fibrotic activities leading to a better recovery post-AKI [67]. 

Considering the possibility of growing the cells under different conditions to improve their biological activity, there are some limitations related to the low yield obtained by cultivating MSCs in two-dimensional (2D) systems (Figure 3). Cao J. et al. attempted to develop a hollow fiber bioreactor-based three-dimensional (3D) device to evaluate the therapeutic efficacy of 3D-derived EVs (3D-EVs) [68]. They cultivated h-UC-MSCs first in 2D flasks and then inoculated them in the 3D bioreactor. 3D-EVs showed the typical exosomal markers and interestingly, they were more concentrated indicating a higher collection proficiency of the 3D system [68]. In a cisplatin-AKI model, 3D-EVs were more effective than 2D-EVs in improving renal function and histology by reducing pro-inflammatory factors, such as the infiltration of T cells and macrophages [68].

### 5.2. Engineering EVs for CKD Treatment 

Engineered EVs were also tested in different models of CKD (Table 7). One of the most interesting strategies is based on the engineering of EVs with Erythropoietin (EPO) [69]. Wang Y. et al. demonstrated the anti-fibrotic capabilities of BM-MSC-derived EVs loaded with EPO (EPO-EVs) in an in vivo UUO-CKD murine model and an in vitro model of TGF-β1-induced fibrosis [70]. In EPO-EV-treated mice, with respect to the naïve MSC-EVs, there was a more evident increase in the number of E-cadherin-positive cells and a decrease of α-SMA positive ones in renal tubules and capillaries [70]. In vitro, EPO-EVs more strongly reversed TGF-β1-induced EMT and inhibited apoptosis in human TECs [70]. Interestingly, in EPO-EVs, there was a greater number of upregulated miRNAs, such as miR-299, miR-499, miR-302, and miRNA-200, which contributed to the biological activity of this EV population both in vivo and in vitro [70]. 

The development of kidney fibrosis is further associated with increased levels of let-7i-5p. To better study the role of this miRNA in the onset and progression of fibrosis, human BM-MSCs were transfected with let-7i-5p antagomir [71]. The BM-MSC-derived EVs were able to transfer the antagomir to NRK-52E cells resulting in the reduction of ECM deposition and the attenuation of EMT [71]. Similar results were obtained in vivo: anti-let -7i-5p was transferred by EVs in the injured kidney of UUO mice inducing a reduction of renal fibrosis and an amelioration of kidney function [71]. The inhibition of let -7i-5p mediated by BM-MSC-EVs induced the activation of the tuberous sclerosis complex subunit 1/mammalian target of rapamycin (TSC1/mTOR) signaling pathway which contributed to the modulation of kidney fibrosis [71]. 

EVs obtained from A-MSCs were transfected with glial cell line-derived neurotrophic factor (GDNF) (GDNF-A-MSC-EVs), which is known to promote the MSC therapeutic effects in renal injury [72]. GDNF-A-MSC-EVs were injected in a murine model of UUO and employed in an in vitro model of hypoxia/serum deprivation (H/SD) injury induced in endothelial cells [73]. In UUO mice, GDNF-A-MSC-EVs reduced peritubular capillary rarefaction and renal fibrosis; in vitro, they stimulated migration, angiogenesis, and resistance to apoptosis to counteract H/SD injury [73]. GDNF-A-MSC-EVs enhanced SIRT1 signaling, which was accompanied by increased levels of phosphorylated endothelial nitric oxide synthase (p-eNOS) [73]. 

EVs were isolated from human A-MSCs which were incubated with melatonin (Exocue), an immune regulator able to prevent tissue fibrosis [74]. Exocue showed an increased expression of some anti-inflammatory and anti-fibrotic miRNAs, as miR-29b-3p, miR-7a-3p, let-7b-5p, let-7c-3p and miR-153-3p [74]. Exocue were injected in CKD mice fed with a 0.25% adenine-containing diet and induced a reduction of renal fibrosis and of the expression of pro-inflammatory cytokines, such as TNF-α and TGF-β [75]. Exocue down-regulated those miRNAs known to be involved in CKD progression, as miR-4270, miR-4739, miR-636, miR-320c, and miR-572, and on the other hand, increased the expression of aquaporin 2 (AQP2) and 5 (AQP5). This resulted in the improvement of kidney function [75].

Bioinformatic analysis predicted the ability of miR-16-5p to target VEGF-A [76]. Human h-USCs were induced to overexpress miR-16-5p by lentiviral transfection [76]. The EVs isolated from h-USC-EVs were able to protect the kidney from podocyte damage through the downregulation of VEGF-A in a miR-16-5p dependent manner both in a rat model of DN and in an in vitro model with high glucose-treated podocytes [76].

**Table 7:** Engineering EVs in CKD.

In Vivo Model	EV-Source	EV Isolation Method	EV Route of Administration	EV Biological Effect	References
**UUO mouse model** Ligation of the left ureter and right ureter was not ligated	m-BM-MSCs (30 µg EV/dose) Tail intravenous injection 1 day after the surgery	UC	Two days of EPO incubation with m-BM-MSC and subsequent EPO-EVs isolation	- Improved renal function and histology - Inhibition of EMT - α-SMA downregulation - Upregulation of E-cadherin expression	[70]
**UUO mouse model** Ligation of the left ureter and right ureter was not ligated	h-BM-MSCs (50 µg EV/dose) Intravenous injection twice a week for 4 weeks	GETTM Exosome isolation kit	h-BM-MSC transfected with Let-7i-5p antagomir in presence of lipofectamine	- Attenuation of ECM deposition and EMT - Amelioration of kidney function and histology - Activation of TSC1/mTOR pathway	[71]
**UUO mouse model** Ligation of the left ureter and right ureter was not ligated	h-A-MSCs (1000 µg EV/dose) Tail intravenous injection	UC	h-A-MSC lentiviral transfection with a vector plasmid system for GDNF gene	- Reduction of renal fibrosis and capillary rarefaction - Improvement of angiogenesis - Enhanced SIRT1 signaling	[73]
**CKD mouse model** Mice were fed with 0.25% adenine-containing diet	h-A-MSCs (50-100 µg EV/dose) Tail intravenous injection for 2 weeks	Exosome isolation kit	h-A-MSCs were treated with melatonin (1µM/mL) and subsequentially Exocue were isolated	- Reduction of renal fibrosis - Downregulation of pro-inflammatory cytokines - Downregulation of pro-fibrotic miRNAs - Increased expression of AQP2 AQP5	[75]
**STZ-DN rat model** Intraperitoneal injection of STZ (65 mg/kg)	h-USCs (100 μg EV/dose) Multiple intravenous injections once a week for 12 consecutive weeks	Exo Quick-TC	Lentiviral transfection of h-USCs	- VEGF-A downregulation - Amelioration of podocyte damage	[76]

Abbreviations: ultracentrifugation (UC), unilateral ureteral obstruction (UUO), streptozotocin diabetic nephropathy (STZ-DN), chronic kidney disease (CKD), bone marrow mesenchymal stromal cells (BM-MSCs), adipose mesenchymal stromal cells (A-MSCs), urinary stem cells, epithelial to mesenchymal transition (EMT).

## 6. Future Perspectives

Renal pathophysiology is a complex and multifactorial process and the possibility of applying regenerative medicine to treat kidney diseases is still far more complex, but EV-based therapies appeared a promising cell-free strategy to induce tissue recovery in different models [15]. Convincing results have been obtained about the protective and pro-survival properties of EVs from different sources in different models of AKI. In recent years, several studies have concentrated on the effects of EVs on CKD onset and progression. 

Stem cell therapies, in particular MSC-based ones, aim to counteract AKI and slow CKD progression, but there are some risks that limit their clinical application. On the other hand, EVs are bioproducts acting as paracrine effectors in cell-cell communication and represent an alternative strategy. Nassar W. et al. conducted a phase II/III clinical pilot study to test the therapeutic potential of human cell-free cord-blood mesenchymal stem cells derived extracellular vesicles (CB-MSCs-EVs) in CKD patients with a diagnosis from more than 6 months [77]. Two different routes of administration were used to improve the EV delivery to the kidneys: the first dose was intravenously injected and the second one was an intra-renal administration [77]. With respect to placebo-treated patients, the EV-treated ones showed a slight reduction of plasma levels of BUN and creatinine and an amelioration of glomerular filtration rate (GFR) [77]. The molecular levels of TGF-β1, a growth factor inducing Treg-mediated immune suppression, were increased in the plasma of patients treated with CB-MSC-EVs [77]. Its increased expression contributed to the downregulation of the pro-inflammatory cytokine TNF-α and the up-regulation of the anti-inflammatory one, IL-10 [77]. These results proved that multiple injections of MSC-EVs were effective in improving the inflammatory response induced by CKD and in protecting kidney histology and function [77].

Despite the encouraging results, more efforts are required for a clinical translation of EVs to treat kidney diseases. There are some achievements to be reached: the upscaling of EV production with adherence to current Good Manufacturing Practices, the standardization of the isolation protocol to reach a higher purity for each preparation, and the need for guidelines for long-period storage. 

## 7. Conclusions

EVs represent a promising therapeutic tool for kidney diseases considering their pro-regenerative, anti-inflammatory, and anti-fibrotic capacities. A meta-analysis study showed that there are no significant associations between the EV biological activity and the cell of origin. Therefore, there are no EV sources that have to be preferred over the others for AKI and CKD treatment [78]. 

Some challenges have to be solved before using EV-based therapies as common medical practice in renal diseases, such as the targeting of specific organ and the loading of therapeutic agents. To enhance EV targeting capacity, researchers are developing EVs with engineered surface proteins. Additionally, the EVs can be endogenously loaded exploiting the cellular machinery to produce the desired cargo molecules sorting them inside EVs [1,2] or through exogenous loading of different chemical compounds and biomolecules directly into the purified EVs [5].

The engineering of EVs has been proposed as a new strategy to enhance their clinical translation. For example, the loading of specific molecules in their hydrophobic core (anti-inflammatory molecules such as IDO and IL-10, specific anti-fibrotic miRNA, EPO, etc.), the modification of the EV-external membrane with targeting moieties to increase EV-biocompatibility (CD47 expression) and the enhancement of their uptake (via P-selectin binding or KIM-1 expression), or again the pre-conditioning of the cell of origin (for example 3D culture conditions, culture in the presence of melatonin, etc.) have been exploited to improve EV-effects. Despite the limitations to be afforded, bioengineered EVs represent a valuable drug carrier for the treatment of kidney diseases in an innovative manner for the next future.

## Figures and Tables

**Table 2:** Non-MSC-EVs in AKI.

In Vivo Model	EV-Source	EV Isolation Method	EV Route of Administration	EV Biological Effect/ Mechanism of Action	References
**IRI rat model** Bilateral renal arterial clamping for 45 min	h-iPSCs and A-MSCs (1 × 10^9^ EV/dose)	UC	Single sub-capsular injection at the beginning of reperfusion in each kidney	- Reduction of inflammation, cell death, and oxidative stress	[39]
**IRI rat model** Clamping of left renal pedicle for 45 min and contralateral nephrectomy	USCs (20 µg EV/dose)	Exo-Quick (co-precipitation)	Single dorsal penis vein injection	- Recovery of renal function - Down-regulation of IRAK1 and inflammation	[40]
**IRI mouse model** Microvascular clamping for 45 min	USCs (20 μg EV/dose)	UC	Single tail intravenous injection 15 min before renal artery clamping	- Regulation of ASCL4-ferroptosis via lncRNA-TUG1	[41]
**Glycerol mouse model** Intra-muscular glycerol injection into inferior hind limbs (8 mL/kg)	Human Urine (2 × 10^8^ EV/dose)	UC	Tail intravenous injection 1 day after the injury	- Inhibition of inflammation - Stimulation of cell proliferation	[42]

Abbreviations: Ultracentrifugation (UC), ischemia and reperfusion injury (IRI), human induced pluripotent stem cells (h-iPSCs), adipose mesenchymal stromal cells (A-MSCs), urinary stem cells (USCs).

**Table 4:** HLSC-EVs in CKD.

In Vivo Model	EV-Source	EV Isolation Method	EV Route of Administration	EV Biological Effect/ Mechanism of Action	References
**STZ-DN mouse model** Intraperitoneal injection of STZ (37 mg/kg) for 4 consecutive days	HLSCs (1 × 10^10^ EV/dose)	UC	Multiple intravenous injections once a week for 4 weeks starting from day 30 after diabetes onset	- Reduced renal fibrosis - Reduced plasma levels of BUN and creatinine - Reduced expression of pro-fibrotic and pro-inflammatory genes	[46]
**AAN mouse model** Intraperitoneal injection of 4 mg/kg of AA on a weekly basis for 4 weeks	HLSCs (1 × 10^10^ EV/dose)	UC	Multiple intravenous injections on a weekly basis starting from 3 days after AA administration	- Reduced tubular necrosis - Reduced interstitial fibrosis - Reduced infiltration of CD45+ cells - Down-regulation of pro-fibrotic genes	[57]
**IRI mouse model** 30 min IRI with contralateral nephrectomy	HLSCs (1 × 10^9^ EV/dose)	UC	Two intravenous injections: one immediately after the surgery and one after three days	- Reduced creatinine plasma levels - Reduced development of interstitial fibrosis - Reduced expression of pro-inflammatory and pro-fibrotic genes - Modulation of genes involved in the EMT process	[44]

Abbreviations: ultracentrifugation (UC), Streptozotocin diabetic nephropathy (STZ-DN), Aristolochic acid nephropathy (AAN), human liver stem cells (HLSCs), ischemia and reperfusion injury (IRI).

**Table 5:** Non-MSC-EVs in CKD.

In Vivo Model	EV-Source	EV Isolation Method	EV Route of Administration	EV Biological Effect/ Mechanism of Action	References
**IRI mouse model** 35 min IRI and contralateral nephrectomy	m-TECs (200µg EV/dose)	UC	Multiple intravenous injections after IRI and continued every 12 h for seven times	- VEGF-A induced cell proliferation - Impairment of AKI-CKD transition	[58]
**STZ-DN rat model** Intraperitoneal injection of STZ (65 mg/kg)	h-USCs (100 μg EV/dose)	UC with Ultra-clear tube (Merck-Millipore, Darnstadt, Germany)	Multiple tail intravenous injections once a week	- Decreased albuminuria - Increased CD31+ cell proliferation - Reduced podocyte apoptosis	[59]

Abbreviations: ultracentrifugation (UC), ischemia and reperfusion injury (IRI), streptozotocin diabetic nephropathy (STZ-DN), murine tubular epithelial cells (mTECs), human urinary stem cells (h-USCs), proximal tubular cells (PTCs).

**Table 6:** Engineering EVs in AKI.

In Vivo Model	EV-Source and Route of Administration	EV Isolation Method	EV Engineering Method	EV Biological Effect	References
**IRI mouse model** Left renal pedicle clamping for 15, 30, and 45 min	P-MSCs (100 µg EV/dose) Intravenous injection after 12 h from reperfusion	UC	Hydrophobic insertion of P-Selectin Binding Protein (PBP) coupled with Cyanine 5.5	- Nephroprotection - Improvement of angiogenesis - Repair of renal parenchyma	[61]
**Cisplatin mouse model** Subcutaneous cisplatin injection (10 mg/kg)	h-UC-MSCs and neutrophils (1 × 10^10^ EV/dose) Tail intravenous injection for 2 days	-h-UC-MSC-EVs obtained via UC -Neutrophil-EVs obtained via cold ultrasonication	NEX obtained from ratio 1:1 of h-UC-MSC-EVs and neutrophil-EVs via sequential polycarbonate membrane extrusion	- Reduction of inflammation - Inhibition of apoptosis and induction of proliferation - CD47-mediated inhibition of macrophage uptake	[62]
**IRI mouse model** Ligation of left renal artery and vein with contralateral nephrectomy	TECs (100 mg EV/0,5 mL) Single tail intravenous injection 12 h after the surgery	UC	Adenovirus transfection of TECs inducing CD26 overexpression	- Reduction of inflammatory cell infiltration by modulation of CXCR4	[63]
**IRI mouse model** Renal pedicle clamping for 25 min	h-BM-MSCs (100 µg EV/dose) Intravenous injection 6 h after IRU	UC	Plasmid transfection to overexpress IDO	- Reduction of inflammatory infiltrates - Inhibition of macrophage polarization	[64]
**IRI mouse model** Renal pedicle clamping for 35 min	RAW macrophages (200 µg EV/dose) Intravenous injection after reperfusion and every 24 h three times	UC	CMV-MCSSV40-Neomycin IL-10 plasmid transfection with Lipofectamine	- Reduction of macrophage infiltration - Attenuation of AKI-CKD transition - Suppression of mTOR and improvement of mitophagy	[65]
**IRI and UUO mouse model** IRI: Renal pedicle clamping for 35 min UUO: ligation of the left ureter and right ureter was not ligated	Red blood cells (100–150 µg EV/dose) Multiple injections once a day for five consecutive days	UC	Engineering of EV surface with Kim-1 bound to LTH peptide and EV cargo with siRNA for P65 and Snail65	- Alleviation of tubulointerstitial inflammation - Attenuation of AKI-CKD transition	[66]
**IRI mouse model** Renal pedicle clamping for 40 min	P-MSCs (100 µg EV/dose) Intravenous injection into three sites 5 min after reperfusion	UC	Naïve EV combined with collagen matrix	- Induction of renal cell proliferation - Stimulation of pro-angiogenic and anti-fibrotic activity	[67]
**Cisplatin mouse model** subcutaneous cisplatin injection (18 mg/kg)	h-UC-MSCs (100 µg EV/dose) Two intravenous injections at 24 and 48 h	UC	Bioreactor 3D	- Improvement of renal function - Reduction of inflammation	[68]

Abbreviations: ultracentrifugation (UC), ischemia and reperfusion injury (IRI), unilateral ureteral obstruction (UUO), placental mesenchymal stromal cells (P-MCSs), umbilical cord MSCs (UC-MSCs), tubular epithelial cells (TECs), bone marrow mesenchymal stromal cells (BM-MSCs), acute kidney injury to chronic kidney disease (AKI-CKD), Interleukin 10 (IL-10).

## Data Availability

Not applicable.
